# Examining Mental Health, Academic, and Economic Stressors During the COVID-19 Pandemic Among Community College and 4-Year University Students

**DOI:** 10.1177/00915521231163929

**Published:** 2023-05-10

**Authors:** Amaranta Ramirez, David B. Rivera, Adrian M. Valadez, Samantha Mattis, Alison Cerezo

**Affiliations:** 1 University of California Santa Barbara, USA

**Keywords:** Coronavirus, COVID-19, community college students, mental health, economic stress

## Abstract

**Objective:** The COVID-19 global pandemic has created severe, long-lasting challenges to college students in the United States (US). In the present study, we assessed mental health symptomatology (depression, anxiety, life stress), academic challenges, and economic stress during the first wave of the Coronavirus pandemic. **Method:** A total sample of 361 college students (*M*_age_ = 22.26, *SD* = 5.56) was gathered from a community college (*N =* 134) and mid-size public university (*N =* 227) in Southwest US, both designated as Hispanic Serving Institutions. **Results:** Pearson and point biserial correlations indicated associations between mental health symptomatology, academic challenges, and economic stress, including expected delays in graduation. Multivariate analysis revealed that community college students had statistically significantly higher scores on anxiety *F*(1, 312) = 5.27, *p* = .02, $\eta_{p}^{2}$ = .01 than 4-year university students, as well as key differences with respect to academic challenges. Chi Square analyses revealed that Latinx families experienced greater economic hardships, including job loss or reduced work hours (χ^2^ (1, *N* = 361) = 28.56, *p* = .00) than other ethnic/racial groups. **Conclusions/Contributions:** Findings revealed that community college students faced disparately negative mental health symptomatology, academic challenges, and economic stress during the first wave of the Coronavirus pandemic. Further, Latinx students’ families experienced significant economic hardship that may have impacted students’ academic progress and future planning.

The COVID-19 global pandemic upended higher education institutions, forcing many campuses to temporarily close and placing many undergraduate students in the precarious position of not knowing what the future holds. A key response to COVID-19 was the implementation of widespread shelter-in-place orders to slow the spread of the virus, inadvertently increasing social isolation for many students for whom social and academic networks on campus are integral to their mental and academic health. In the general US population, COVID-19 demonstrated unprecedented detrimental effects on stress, anxiety, and loneliness (Guntuku et al., 2020). Among college students in the US and abroad, COVID-19 has been associated with anxiety and depression (Chirikov et al., 2020; Liu et al., 2020) as a result of daily life stress, disruptions to academic activities, and economic stress that many students have been forced to navigate (Cao et al., 2020; Hoyt et al., 2021).

The disparate effect of COVID-19 among ethno-racial minority and community college students is an important area of research that only just begun to emerge. Across the US, Latinxs, and African Americans have been disparately negatively affected by COVID-19 contraction, hospitalization, and death (Centers for Disease Control & Prevention, 2020, 2021; Macias Gil et al., 2020; New York Department of Health, 2020) in addition to loss of income and economic stability (Clay & Rogus, 2021). Further, community college students consistently face greater barriers to their higher education pursuits than 4-year university students. For instance, researchers analyzing pre-COVID-19 data discovered that community college students encountered greater odds of depressive and anxious symptoms than 4-year university students (Lipson et al., 2021). Yet, limited data on the differential impacts of COVID-19 by race and ethnicity and between community college and 4-year university students have not yet been examined (Hoyt et al., 2021).

As the pandemic continues to unfold, infection, hospitalization, and mortality rates continue to climb as new variants spread across the US. It remains unclear how COVID-19 will continue to impact US higher education systems in the years to come. It is therefore critical to understand how pandemic conditions, including at the onset of city and statewide mandates, influence college students’ mental health as well as their academic and economic demands.

## COVID-19 and Its Impact on Mental Health

COVID-19 has been shown to have significant mental health effects on college students. Active Minds (2020) sampled 2,086 college students in April 2020 and found that 91% endorsed stress or anxiety, 81% endorsed sadness or disappointment, and 80% endorsed loneliness or isolation in relation to COVID-19. Further, 80% of college students said that their mental health either worsened or worsened significantly as a result of COVID-19. Among a college student sample recruited by Hoyt et al. (2021), cisgender women met the threshold for moderate anxiety and perceived stress between April 2020 and July 2020. In a longitudinal study of US college students, Huckins et al. (2020) found that anxiety, depression, and sedentary time all increased significantly at the start of the national declaration of emergency as compared to previous quarters. Similarly, Chirikov et al. (2020) sampled 30,725 undergraduates and 15,346 graduate students across nine public universities in the US. The authors found that twice as many students screened positive for major depressive disorder and 1.5 as many students screened positive for anxiety disorders during May to July 2020 as compared to 2019 prevalence rates.

The COVID-19 global pandemic brought extreme changes to everyday functioning and socialization. Reports indicated that 52% of young adults moved in with family members for cohabitation during the extended lockdowns (Pew Research Center, 2020), with the majority being college students whose campuses closed. Forty-nine million people were laid off or temporarily lost employment (U.S. Department of Labor, Bureau of Labor Statistics, 2020), and schools throughout the US were forced to shut down. For college students in particular, school closures meant shifting to remote online learning. Given these abrupt changes to daily life, the social distancing mandate, and changes to learning, college students faced increased risk of developing new or worsening mental health problems including depression, substance misuse, and suicidal ideation (Czeisler et al., 2020). National pre-COVID-19 data show concerning trends in mental health symptomology among college students as compared to previous years (Duffy et al., 2019). The distressing circumstances brought about as a result of COVID-19 call for a greater need to better understand how students in both community colleges and 4-year universities have been affected.

Given the limited known effects of COVID-19 among college students at the time of this study, the present study’s hypotheses were exploratory in nature. Consistent with California and national trends, our first hypothesis was that Latinx students would report higher rates of negative economic and mental health symptomatology than their ethno-racial peers during the first wave of the pandemic, which was defined as the beginning of the first pandemic lock-down in March of 2020 (CalMatters, 2020). While the economic fallout of Coronavirus was ubiquitous, in May 2019, 59% of Latinxs experienced job losses or pay cuts due to Coronavirus as compared to 43% of the general US population (Pew Research Center, 2020). In fact, the highest rate of job loss among all ethno-racial and gender groups in the US was among Latinas (Pew Research Center, 2020). We therefore hypothesized that Latinx students would report the greatest economic stress as a result of COVID-19 and that relatedly, they would also report higher mental health symptomatology. Our second hypothesis was that community college students would face a higher rate of economic stress than 4-year university students. This hypothesis was influenced by the extant economic differences between community college and 4-year university students (Smith, 2019). Finally, we hypothesized that campus closures would have an adverse effect on university and community college students across the board. Given the sudden shift to online based learning and reduced access to resources like tutoring or technology, we hypothesized that all students would experience academic challenges.

## Method

### Recruitment

The goal of the present study was to assess mental health symptomatology, academic challenges, and economic stressors among college students during the first wave of the Coronavirus pandemic. Participants were sampled from a community college (36.8%) and mid-size public university (63.2%) in Southern California. Data were collected between May 20 and May 30, 2020. The survey was closed on May 30, 2020 at the start of protests in Southern California in response to the death of George Floyd. It is important to note that shelter-in-place began on March 19, 2020 in California (CalMatters, 2020). Thus, the present study reflects the immediate impacts of COVID-19 and shelter-in-place mandates on college students.

Upon securing IRB approval, participants were recruited from a community college and a mid-size public university in Southern California, both designated as Hispanic Serving Institutions. Recruitment advertisements were sent out by the research team to department listservs, student groups, and an online network of community college counselors in Southern California. The recruitment flyer detailed the participation criteria as anyone who was a college student and 18 years of age or older in Southern California. Participants were given the opportunity to join a lottery to win a $20 Amazon gift card for participation in the study.

The aim of the present study was to recruit a diverse sample of college students in Southern California. Recruitment solely occurred in Southern California given the unique socio-economic and racial-ethnic composition of the region. In 2019, prior to the start of Coronavirus, the median household income in Los Angeles was $68,044 and Latinxs comprised 48.6% of the population.

### Participants

On average, participants were 22 years old (*M* = 22.26, *SD* = 5.56) with 36.8% enrolled in a community college and 63.2% enrolled in a 4-year public university. Community college students were an average of 23 years old, while four-year university students were 21 years old. The majority of the sample identified as cisgender women (67.3%). In terms of race and ethnicity, 36.3% of the sample identified as Latinx, 28% as White, 18% as Asian American, 8.3% as Multiracial, 6.1% as Other unspecified, and 3.3% as African American.

The sample reflects the ethno-racial diversity of the campuses from which participants were recruited. For the community college, the ethno-racial breakdown of the larger student body is 35.8% Latinx, 30% White, 10.6% Asian/Pacific Islander, and 7.5% Black. For the university, the ethno-racial breakdown of the larger student body is 28% Latinx/Chicano, 38% White, 27% Asian/Pacific Islander, and 6% Black/African American/Other. Given that a central goal of this study was to examine differences by ethno-racial groups, the last three groups (Multiracial, Other unspecified, and African American) were collapsed into a single “Other Underrepresented Minority (URM)” as a means to retain them in the study since respective number of participants per group was too small to permit individual analyses. Together, Other URM students comprised 17.7% of the sample.

Related to sexual orientation, 79.5% of participants identified as heterosexual, 8% as queer, pansexual or other, 7.8% as bisexual, and 4.7% as gay/lesbian. With respect to economic standing, 77% of participants reported a family income that fell under the California poverty line ($97,200 for a family of four; Health for California, 2021). Twenty-five percent of participants reported being unemployed, and family unemployment was reported among 32.1% of the sample. A large majority of participants reported having health insurance (91.7%). However, 32% of participants reported that they did not have or were not sure if they had a primary care physician. See Table 1 for additional demographic information.

**Table 1.** Correlations for the Full Sample.

	1.	2.	3.	4.	5.	6.	7.	8.	9.
1. Anxiety	—								
2. Depression	.72**								
3. Life stress	.45**	.48**							
4. Job impact—family	.16**	.13**	.10						
5. Job impact—self	.14**	.14**	.13*	.18**					
6. Difficulty concentrating	.09	.26**	.13*	−.00	−.02				
7. Computer access	.12**	.07	.06	.10*	.14**	−.02			
8. Internet access	.13**	.12**	.05	.10*	.08	.08	−.01		
9. Delayed graduation	.19**	.17**	.14**	.14**	.05	.02	.01	.06	—

* *p* < .05. **p < .01.

### Measures

The present study used a cross-sectional design with a convenience sample of college students’ representative of colleges and universities in Southern California. Several measures were used to assess participants’ experiences of mental health symptomatology (depression, anxiety, and life stress). We also developed measures specific to academic and technology challenges and economic stressors that were linked to COVID-19 and consequent shelter-in-place mandates. Participants were administered a single instrument containing these measures.

#### Questionnaire

The demographic section of the questionnaire included items about age, race and ethnicity, gender identity, sexual orientation, school of attendance, education level, health insurance status, economic stressors, and healthcare access. Participants were also asked whether their employment and that of their family members was impacted by COVID-19.

Depressive symptomatology was measured using the Center for Epidemiologic Studies Depression Scale Revised-10 (CESD-R 10; Bjorgvinsson et al., 2013). The CESD-R 10 is a 10-item scale that screens for depression and depressive disorder by measuring symptoms defined in the DSM-V for major depression. Sample items include, “I had trouble keeping my mind on what I was doing” and “I felt that everything I did was an effort.” Items were rated using a 4-point Likert-type scale from 0 = “Rarely or none of the time” to 3 = “All of the time.” The total score on the CESD-R 10 was used in the present study. Inter-item reliability for this sample was α = .83

Anxious symptomatology was assessed using the General Anxiety Disorder-7 (GAD-7) scale (Spitzer et al., 2006). The GAD-7 is a 7-item scale that screens for general anxiety disorder, measuring symptoms, and functional impairment. Sample items include, “Feel nervous, anxious or on edge” and “Worrying too much about different things.” The GAD-7 uses a 4-point Likert-type scale ranging from 0 = “Not at all” to 3 = “Nearly every day.” The total score for the GAD-7 was used in the present study. Inter-item reliability for this sample was α = .91.

Life stress was measured using the Stress in Context (SIC) scale (Mayer et al., 2017). The SIC is a 21-item scale that assesses perceptions of stress in specific contexts and includes items like, “Feeling like you have control over your life means that you can determine how you spend each day. How often do you feel like you have a lot of control over your daily life?” and “Having a life that is uncertain means that bad things can happen to you at any time. How often do you feel that your life is uncertain?” The SIC is rated on a 4-point Likert scale ranging from 1 = “Never” to 4 = “Usually.” In this study we modified the SIC questionnaire to only include the first 17 questions of the scale, with the last three questions excluded because they focus on childhood experiences not relevant to COVID-19. Inter-item reliability for this sample was α = .67. The original measure has an inter-item reliability of .77 (Mayer et al., 2017).

Academic challenges were assessed by asking participants whether online classes impacted their ability to succeed, if they anticipated graduation delays, and their access to technology and internet as a result of COVID-19. Specifically, we asked, “How has the switch to online classes affected your ability to succeed in school?” Participants responded to one of two options that included, 1 = “It’s difficult for me to complete assignments” and 2 = “I haven’t had any difficulties.” We also asked, “Has COVID-19 impacted your graduation plans?” with two response options being 1 = “No,” and 2 = “Yes.” With respect to technology issues, we asked participants: “Have you faced any challenges with technology or the internet?” Participants responded to one of three options: 1 = “No, I have not faced any challenges, 2 = “Yes, I sometimes don’t have access to a computer,” and 3 = “Yes, I sometimes don’t have reliable internet.”

Economic stress was measured through a series of questions regarding how COVID-19 affected participants’ own employment and their family’s employment status. The following questions were posed to participants. “Are you currently employed?,” to which participants could respond either 1 = “No” or 2 = “Yes.” Two additional questions specifically focused on the effects of COVID-19 on employment; “Have you lost your job, or had hours reduced due to COVID-19?” and “Has someone in your family lost their job or had hours reduced due to COVID-19?.” Participants were able to choose one of three responses: 1 = “No,” 2 = “Yes, I have lost my job (Yes, they have lost their job),” and (3) “Yes, my hours were reduced (Yes, their hours were reduced).”

### Procedure

The survey was hosted online via Qualtrics.com. Each participant created an original username. Following completion of the survey, participants had the option of emailing their username to the study team’s email address provided at the end of the survey to be entered into a drawing to win a $20 Amazon gift card. A total of 533 responses were initially collected and a final sample of 361 was retained. Participant data were not retained if there was more than 20% missing data (Schlomer et al., 2010), if the survey was completed in less than 400 s (6.5 min), and if participants did not respond to security items correctly (e.g., “For security purposes, please type the number 14 into the textbox”). The questionnaire was previously vetted by the researchers for the length of time it took to complete, with the average of 12 to 15 min. Participants who finished the survey in under 6.5 min were considered false respondents.

### Data Analysis

Prior to running correlations and group comparisons, data were screened to ensure that the assumption of normality was met for the proposed statistical analyses. Measures for depression (Skewness = 0.118; Kurtosis = −0.59), anxiety (Skewness = 0.28; Kurtosis = −0.83), and life stress (Skewness = −0.35; Kurtosis = 0.67) demonstrated that normalcy assumptions were met. Scores between −2.0 and 2.0 were considered to be within acceptable bounds for skewness while scores of −7.0 and 7.0 were within acceptable bounds for kurtosis (Curran et al., 1996). Next, we examined associations between key variables via Pearson and point biserial correlations which allowed for correlational analysis between continuous and dichotomous variables. Following correlations, One-Way MANOVA (continuous data; Weinfurt, 1995) and Chi-Square (categorical data; Singhal & Rana, 2015) were used to test for group differences between ethno-racial groups and between students enrolled in a 4-year university or community college. Consistent with California and national trends, we hypothesized that Latinx students as well as community college students would face higher incidence of academic and economic challenges during COVID-19.

## Results

For the full sample, participants reported elevated depressive symptomatology (*M* = 14.37, *SD* = 6.31), on average scoring higher than the suggested cut off of 10 points for symptomatic depression (Zhang et al., 2012). Participants’ average score of anxiety (*M* = 9.91, *SD* = 5.9) met the criteria for mild to moderate symptomatic anxiety (Spitzer et al., 2006).

### Correlations

Correlation analysis across key study variables were run for the full sample. It is important to note that mental health scores demonstrate symptomatology between May 20 and May 30, 2020 with participants indicating their emotional and behavioral symptomatology for the previous 2 week. Data were not intended to indicate trait scores of mental health. Rather, the findings reported indicate mental health symptomatology during a period of extreme stress and uncertainty. Further, both depression and anxiety symptomatology were positively associated with life stress, job loss for oneself, job loss for one’s family, and expected delays in graduation. See Table 1 for correlation results among study variables.

### Mental Health Symptomatology

#### Outcomes by race/ethnicity

A one-way MANOVA was conducted to examine differences in reported symptomatology in the areas of depression, anxiety, and life stress between ethno-racial groups. Categories were: Latinx (*N* *=* 131), White (*N* *=* 101), Asian American (*N* *=* 65), and Other URM (*N* *=* 64). Box’s Test of Equality of Covariance Matrices indicated that groups were not significantly different from each other (*p* = .59), meeting normality assumptions. Multivariate tests showed non-significant effects by race/ethnicity (Pillai’s trace = 0.059, *F*(4, 321) = 1.62, *p* = .078, $\eta_{p}^{2}$ = .02).

#### Outcomes by college-type

A one-way MANOVA was conducted to examine differences across depression, anxiety, and life stress by college type (see Table 2). Multivariate tests showed significant effects by college type (Roy’s largest root = 0.001, *F*(4, 308) = 4.84, *p* = .001, $\eta_{p}^{2}$ = .59). Tests of between subjects’ effects showed that anxiety (*F*(1, 312) = 5.27, *p* = .022, $\eta_{p}^{2}$ = 0.01) had a significant effect by college type while depression and life stress did not. Thus, community college students experienced statistically significant higher rates of anxiety than 4-year university students.

**Table 2.** Depression and Anxiety Symptomatology Compared to National Averages.

Mental health symptomatology	Present study	Duffy et al. (2019)
Depression	73.7%	23%
Anxiety	46.3%	21.4%

### Academic and Economic Challenges

#### Outcomes by race/ethnicity

A Chi-Square test of independence was conducted to test the relationship between race/ethnicity and whether the switch to online learning impacted students’ ability to succeed in school. The relationship was not significant (χ^2^(1, *N* = 361) = 6.34, *p* = .10). Across the board, the switch to online learning appeared to negatively affect all students. In fact, a large majority of Asian American (83.1%) and Hispanic/Latinx (80.2%) respondents reported inability to succeed in school, followed by 75% of Other URM students and 68.3% of White students. A Chi-Square test of independence was also conducted to test the relationship between race/ethnicity and expected delay in graduation. The relationship was not significant (χ^2^(3, *N* = 361) = 6.84, *p* = .08). Results showed that Latinx students reported the highest rate of expected delay in graduation (35.9%) followed by students in the Other URM category (34.4%), White students (28.7%), and Asian American students (18.5%).

A Chi-Square test of independence revealed no significant relation between race/ethnicity and access to a computer (χ^2^ (1one, *N* = 361) =6.32, *p* = .10). Most respondents indicated having access to a computer with highest access among White students (97%) followed by Asian American students (93.8%), Other URM students (87.5%) and Latinx students (88.5%). A Chi-Square test of independence also revealed no significant relation between race/ethnicity and lacking consistent access to the internet (χ^2^(1, *N* = 361) = 7.35, *p* = .06). The highest rate of lacking access was experienced by Other URM students (57.8%) followed by Latinx students (53.4%), White students (43.6%), and Asian American students (40%).

**Figure 1.**
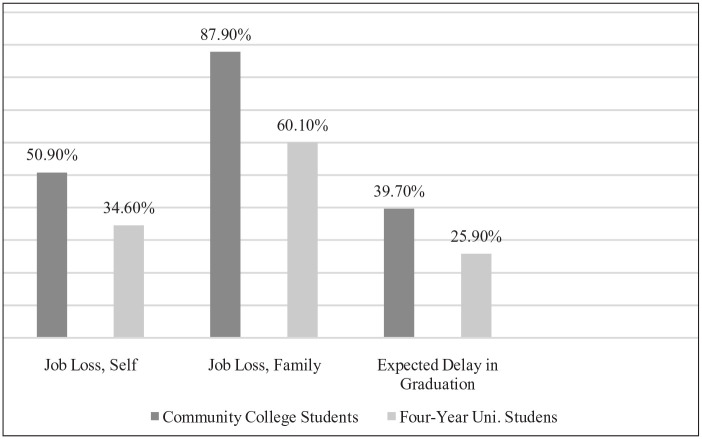
Important economic and academic stressor differences by college-type.

A Chi-Square test of independence was conducted to test the relationship between race/ethnicity and family job loss and/or reduction in work hours (see Figure 1). The relationship was significant (χ^2^(1, *N* = 361) = 28.56, *p* = .00). Findings demonstrated that Latinx students’ families experienced the highest employment impacts with 84.4% reporting job loss and/or reduced hours. Next, 75% of Other URM students reported job loss and/or reduced hours followed by 57.4% of White and 55.4% of Asian American respondents. A Chi-Square test of independence was also conducted to test the relationship between race/ethnicity and personal job loss and/or reduction in work hours. The relationship was not significant (χ^2^(1, *N* = 361) = 7.48, *p* = .06). The highest rates of personal job loss and/or reduction in work hours was experienced by Other URM students (48.4%) followed by Latinx students (44.3%), White students (36.6%), and Asian American students (27.7%).

#### Outcomes by college-type

A Chi-Square test of independence was conducted to test the relationship between college type and whether the switch to online learning impacted students’ ability to succeed in school. The relationship was not significant (χ^2^(1, *N* = 361) = 6.34, *p* = .10). A high proportion of both groups reported negative impacts; 78.9% of community college students and 76.8% of 4-year university students. A Chi-Square test of independence was also conducted to test the relationship between college type and expected delay in graduation. The relationship was significant (χ^2^(3, *N* = 361) = 6.88, *p* = .01). Results showed that 39.7% of community college students reported a delay in graduation plans compared to 25.9% of 4-year university students. Thus, community college students were 1.9 times more likely to report a delay in graduation plans.

A Chi-Square test of independence revealed no significant relation between college type and access to a computer (χ^2^(1, *N* = 361) = 3.34, *p* = .07). However, findings revealed that 11.2% of community college students had no access to a computer, contrasted to 5.7% of 4-year university students. A Chi-Square test of independence also revealed no significant relation between college type and lacking consistent access to the internet (χ^2^(1, *N* = 361) = 2.33, *p* = .13). That said, a greater proportion of community college students reported trouble accessing the internet (54.3%) compared to 4-year university students (45.6%).

A Chi-Square test of independence was conducted to test the relationship between college type and family job loss and/or reduction in work hours. The relationship was significant (χ^2^(1, *N* = 361) = 28.11, *p* = .00). Findings showed that 87.9% of community college students reported that a family member had lost their job or had their hours reduced compared to 60.1% of 4-year university students. A Chi-Square test of independence was also conducted to test the relationship between college type and personal job loss and/or reduction in work hours. The relationship was significant (χ^2^(1, *N* = 361) = 8.41, *p* = .00). Findings showed that 50.9% of community college students reported that they themselves had lost their job or had their hours reduced compared to 34.6% of 4-year university students.

## Discussion

The findings of the present study confirm and expand extant research on the challenges faced by college students in the US with respect to mental health symptomatology, academic challenges, and economic stress during the COVID-19 pandemic. Similar to other studies (Cao et al., 2020; Chirikov et al., 2020; Liu et al., 2020), participants in our sample indicated high scores in the areas of depression and anxiety symptomatology. These scores indicate concerning levels of mental health symptomatology in college students in May 2020, the start of campus closure and shelter-in-place mandates.

Further, the rates of depressive and anxious symptoms present in this sample were higher when compared to pre-Coronavirus national samples across the US (Duffy et al., 2019). While recent pre-Coronavirus national data suggested an increase of mental health challenges, with 14% to 23% of students reporting symptoms of depression (Active Minds, 2020), 73% of participants in the present study reported depressive symptomatology and 46.3% reported anxiety symptomatology in response to COVID-19. The higher rates of depressive and anxious symptomatology revealed within the presented study confirm other college student COVID-19 mental health research findings (Active Minds, 2020; Healthy Minds Network, 2020).

A key goal of the present study was to examine group differences in the presentation of mental health symptomatology, academic challenges, and economic stressors by race/ethnicity and college type (4-year university vs. community college). Our findings demonstrated no statistically significant differences in mental health symptomatology by race/ethnicity. Rather, students across the board reported high levels of depression and anxiety symptomatology. With respect to other group differences, data revealed that a higher proportion of Latinx and Other URM students reported having family members lose their jobs and/or face a reduction in work hours than Asian American and White students. These data confirm national trends of economic stress among Latinx workers (Pew Research Center, 2020).

At the time of our study few extant studies of COVID-19 among college students had sampled community college students. In the current study, community college students reported statistically significantly higher scores on measures of anxiety than 4-year university students. Community college students already contend with a shortage in mental health resources with many campuses facing stark cuts to their mental health services prior to COVID-19 (Anderson, 2019). It is therefore critical that additional research on mental health needs of community college students be conducted to address the inadequacy of support resources across community college campuses.

A key finding in our study was that a greater proportion of community college students anticipated a delay in their graduation plans. In October 2020, US Census data (U.S. Census Bureau, 2020) showed that community college students were canceling their plans to attend college at more than twice the rate of 4-year university students. Further, the US Census report showed that about 40% of community college students reported experiencing job loss since March 2020, and noted that loss of income was one of the key reasons for changing their college plans. These trends were confirmed in the present study where 87.9% of community college students reported losing a job and/or seeing a reduction in work hours in addition to 50.9% of students’ families losing a job and/or seeing a reduction in work hours. These findings demonstrate severe economic impacts on community college students in Southern California in May 2020 in relation to COVID-19, likely impacting their future educational and career aspirations.

### Strengths and Limitations

The current study demonstrated several noteworthy methodological strengths and contributions. This study is novel in that it contributes to a limited body of work focused on the experiences of community college students during the COVID-19 shelter-in-place order. While this demographic was not the sole focus of the study, the representation of community college students in the sample allowed for a closer examination into the needs of these students and how they might differ from those at 4-year institutions. The use of Hispanic Serving Institutions in participant recruitment afforded greater inclusion and diversity compared to other convenience sampling methods. This study’s sample closely reflects the racial-ethnic student composition of the community colleges and 4-year institutions where recruitment occurred.

Another strength of our study was that the background demographic survey questions were intentionally developed to allow for a diverse representation of our participants’ experiences related to COVID-19. Specifically, we developed items related to academic challenges and economic stress specific to COVID-19, capturing the immediate impact of shelter-in-place on students and their families.

This study also included several limitations important to consider. A cross-sectional design was used which means that causal claims were unable to be made. A longitudinal study would provide additional information about the long-term effects of COVID-19 and the associated stay-at-home orders. Additionally, our sample was comprised of a low number of Multiracial and African American respondents, and zero respondents that self-identified as Native American. Preliminary analysis was run on the key mental health scores to ensure no statistically significant differences existed between these groups, thus allowing their resulting placement into an “Other URM” category. However, collapsing ethnic/racial groups into an “Other URM” category likely obfuscated important group differences. Finally, the George Floyd protests across the United States that began May 26, 2020 may have posed a potential confounding factor to measures of anxiety, depression, and life stress. While recruitment closed prior to protests reaching Southern California, we would be remiss if we did not acknowledge that national media attention, including social media, may have impacted students’ mental health symptomatology.

### Recommendations for Research and Clinical Practice

Several recommendations for academia and clinical practice follow from the present study findings. First, higher education institutions should consider that particular student groups may experience disparate challenges navigating the effects of the ongoing pandemic (see Active Minds, 2020; Conrad et al., 2021; Kim et al., 2022). In the present study, we found important differences by college type and between ethnic/racial groups related to employment and economic stress. Higher education institutions—particularly community colleges—should increase efforts to connect students with supplemental resources (e.g., virtual tutoring, financial resources, technological/troubleshooting support, etc.) early into their courses to prevent attrition, decreases in GPA, and/or delays in graduation. This is especially critical given the ongoing incidence of Coronavirus variants and their potential impact on campus operations and potential future closures (see United Nations Educational, Scientific and Cultural Organization, n.d).

As demonstrated by our findings, it is possible that while certain academic institutions have attempted to accommodate the technological needs of their students during the COVID-19 pandemic (see Los Angeles Community College District, 2020), information related to these resources may not be reaching students most in need. A potential consideration may be for instructors to list resources (e.g., food bank, cash aid) on their course syllabi and/or via repeated verbal announcements in class, and to also list these resources on the online platform where their courses are made available to students. Considering that technology challenges were more frequently reported among community college students, programs which aim to serve culturally diverse students at community colleges (e.g., the Educational Opportunities Program [EOP]) should consider ensuring that students have access to adequate technological resources to thrive in their courses. Further, community college students reported significant adverse effects to their employment status. Academic institutions should actively recruit students to federal, state, and county supports like *The California Work Opportunities and Responsibility to Kids* (CalWORKs), which provide financial support via books, tuition and in some cases, housing assistance and health insurance for student parents.

The study findings on mental health symptomology amidst COVID-19 suggest implications for university and community college counselors and clinicians. University and community college counselors and clinicians should be increasingly conscious of co-occurring symptoms of comorbidities (e.g., the presence of symptoms of both depression and anxiety) among students as this may impact potential treatment(s). Students reported heightened symptomatology in the areas of depression, anxiety, and life stress with some of these symptoms more pronounced among Latinx and Other URM students. As such, university counselors and clinicians should consider the role of each student’s unique cultural background given ongoing disparities in California and nationally. In regard to additional support resources for community college students, counselors, and clinicians can help students connect with outside providers and organizations that provide services at no or reduced cost. This includes non-profits, community clinics, and online supports like CalHOPE, which provides crisis counseling during national emergencies (AACC, 2023).

Results from the present study demonstrate that more research is needed to examine the unique effect of the COVID-19 pandemic on the mental health of students, with clear disparities affecting community college students. To explore key within-group differences of the impact of COVID-19, researchers should collect qualitative data via individual interviews and/or focus groups. These data would be key to developing tailored interventions that account for the long-term and differential impact of COVID-19 across unique student populations.
